# Supplementary material for: Content-rich biological network constructed by mining PubMed abstracts
Source: BMC Bioinformatics. 2004 Oct 8;5:147. doi: 10.1186/1471-2105-5-147 (PMC528731; doi:10.1186/1471-2105-5-147)
Supplement: Additional File 5 — The original Chilibot query results of the term "long-term potentiation (LTP)" and 22 other terms, limiting the latest references analyzed to the years 1990, 1995, 2000, and 2004. [file 1471-2105-5-147-S5.bz2 › chilibotAdditionalFile5/ltp2000/html/PKC_NMDA.html]

 


 **PKC** and **NMDA** 
  
Found 166 abstracts in PubMed,  **30 abstracts were retrieved and analyzed**.  


---

 Search Google  |
 PDF files only 
|  EDU domain only 

---

**Interactive relationship** (e.g. stimulation, inhibition, etc)

- **NMDA**  currents from Xenopus oocytes expressing recombinant zeta1 epsilon2  **NMDA**  receptors can be potentiated by activation of protein kinase C  **PKC**  and also demonstrate time dependent rundown.  Ref: 10505612 Neurosci Lett, 1999
- The possibility that protein kinase C  **PKC**  is involved in the induction of N methyl D aspartate  **NMDA**  receptor dependent long term potentiation LTP at CA1 synapses in the hippocampus has been the subject of considerable investigation.  Ref: 11069602 Eur J Neurosci, 2000
- In contrast to ACPD induced potentiation no evidence for an involvement of protein kinase C  **PKC**  is found in the CHPG induced potentiation of both  **NMDA**  and AMPA depolarization because the  **PKC**  blockers chelerythrine chloride or calphostin C did not antagonize this effect.  Ref: 10530818 Neuropharmacology, 1999
- Furthermore, the LTP  **NMDA**  deficit in mGluR5 mutant mice could be rescued by stimulating protein kinase C  **PKC**  with 4beta phorbol 1 3 dibutyrate PDBu.  Ref: 10454358 Learn MemLearn Mem, 1999
- NOC oFQ contributed to impaired  **NMDA**  induced pial artery dilation after H I, suggesting, therefore, that  **PKC**  dependent O 2 generation by NOC oFQ links NOC oFQ release to impaired  **NMDA**  dilation after H I.  Ref: 11087221 Am J Physiol Heart Circ Physiol, 2000
- These data show that in LTP K the in situ phosphorylation of pre and postsynaptic  **PKC**  substrates is increased, indicating that  **NMDA**  receptor dependent and  **NMDA**  receptor independent LTP share common calcium dependent expression mechanisms, including activation of pre and postsynaptic  **PKC** .  Ref: 10822151 Neurosci Lett, 2000
- Activation of endogenous  **PKC**  by 4beta phorbol 12 myristate 13 acetate enhanced peak I p and depressed steady state I ss  **NMDA**  evoked currents, resulting in a reduction in the ratio of these currents I ss I p.  Ref: 10844014 J Neurosci, 2000
- NOC oFQ  **PKC**  dependent superoxide generation contributes to hypoxic ischemic impairment of  **NMDA**  cerebrovasodilation.  Ref: 11087221 Am J Physiol Heart Circ Physiol, 2000
- These results suggest that  **PKC**  may couple the postsynaptic mGluR5 to the  **NMDA**  receptor potentiation during LTP,.  Ref: 10454358 Learn MemLearn Mem, 2000
- These results suggest that the increase of calcium influx through  **NMDA**  channels and L type VOCCs in turn triggering a  **PKC**  dependent signaling cascade is a possible cellular basis underlying this seizure like activity induced inhibition of LTP.  Ref: 10980002 J Neurophysiol, 2000
- **PKC**  potentiation of NMDAR function is likely to be an important mode of NMDAR regulation in vivo and may play a role in  **NMDA**  dependent long term potentiation.  Ref: 10611373 Proc Natl Acad Sci U S A, 1999
- The decrease in  **PKC**  activity was prevented by  **NMDA**  receptor blockade and was not accompanied by a decrease in the level of either PKCalpha, beta, gamma, or zeta.  Ref: 11007876 J Neurosci, 2000
- Taken together, these results suggest that  **PKC**  enhances calcium CaM dependent inactivation of the  **NMDA**  channel, most likely because of a phosphorylation dependent regulation of interactions between receptor subunits, CaM, and other postsynaptic density proteins.  Ref: 10844014 J Neurosci, 2000

**Parallel relationship** (e.g. studied together, co-existance, homology, etc.)

- Thus, SP, which is released in the spinal cord subsequent to persistent stimulation of small sensory afferents after tissue injury, may contribute to spinal hyperexcitability and persistent pain by enhancement of  **PKC**  mediated phosphorylation of target molecules such as  **NMDA**  receptors.  Ref: 10986346 Brain Res, 2000
- Protein kinase C  **PKC**  inhibitors H7, staurosporine, GF 209103X, cheleritrine and sphingosine prevented the SRIF 14 effect, while phorbol 12 myristate 13 acetate enhanced the  **NMDA**  response.  Ref: 10821783 Br J Pharmacol, 2000
- Preinjection of oocytes with calyculin A impeded the PKA but not the  **PKC**  mediated potentiation of hypothalamic  **NMDA**  induced currents.  Ref: 10899951 J Neurochem, 2000
- Taxol had no effect on either rundown or  **PKC**  potentiation of  **NMDA**  currents.  Ref: 10505612 Neurosci Lett, 1999
- When the  **NMDA**  channels are open, the determinant role in receptor phosphorylation is played by the  **PKC**  and CaMKII.  Ref: 10885014 Ross Fiziol Zh Im I M Sechenova, 2000
- Because previous results have suggested a role for protein kinase C in insulin potentiation of  **NMDA**  currents in oocytes, the effects of coexpression of PSD 95 on insulin and  **PKC**  potentiation of  **NMDA**  currents from these receptors were compared.  Ref: 10854272 J Neurochem, 2000
- The present study sought to investigate whether  **PKC**  regulates calcium dependent inactivation of the  **NMDA**  channel in hippocampal neurons.  Ref: 10844014 J Neurosci, 2000
- Pb 2 induced cell death was blocked partially by staurosporine  **PKC**  inhibitor, 100 nM and  **NMDA**  antagonist, MK 801 1 microM.  Ref: 10802384 Toxicol Lett, 2000
- **NMDA**  induced currents recorded from XENOPUS oocytes injected with cRNA encoding the  **NMDA**  receptor subunits NR1, NR2A, and or NR2B, mainly found in rat hypothalamus, were not affected by PKA activation but were increased by protein kinase C  **PKC**  stimulation.  Ref: 10899951 J Neurochem, 2000
- It is concluded that SRIF receptors, possibly of the sst 5 subtype, can exert a permissive role on  **NMDA**  receptors colocalized on hippocampal noradrenergic terminals activation of sst 5 receptors is coupled to pertussis toxin sensitive G proteins enhancing phosphoinositide metabolism with activation of InsP 3 receptors and  **PKC** .  Ref: 10821783 Br J Pharmacol, 2000
- This study determined whether nociceptin orphanin FQ NOC oFQ generates superoxide anion O 2 in a protein kinase C  **PKC**  dependent manner and whether such production contributes to hypoxic ischemic H I impairment of N methyl D aspartate  **NMDA**  induced pial artery dilation in newborn pigs equipped with closed cranial windows.  Ref: 11087221 Am J Physiol Heart Circ Physiol, 2000
- The transient increase in the expression of NR2B mirrored the increase in the expression of  **PKC**  sensitive isoforms of the NR1 subunits of the  **NMDA**  receptor we observed previously.  Ref: 10456090 Learn Mem< MedlineTA>Learn Mem, 2000
- Somatostatin potentiates  **NMDA**  receptor function via activation of InsP 3 receptors and  **PKC**  leading to removal of the magnesium block without depolarization.  Ref: 10821783 Br J Pharmacol, 2000
- The MK 801 transient did not alter the ability of  **NMDA**  to trigger normally lethal calcium i influx 48 hr later, but it did block early downstream signaling events coupled to  **NMDA**  neurotoxicity, including  **PKC**  inactivation and the activation of calpain.  Ref: 11007874 J Neurosci, 2000
- We demonstrate that hypoxia leads to  **PKC**  mediated activation of  **NMDA**  receptors in the brainstem,.  Ref: 10643790 Neurosci Lett, 2000
- The results indicate that both spontaneous rundown and  **PKC**  potentiation of currents from heterologously expressed zeta1 epsilon2  **NMDA**  receptors depend on dynamic actin polymerization depolymerization but do not involve changes in microtubules.  Ref: 10505612 Neurosci Lett, 1999
- In addition, we show that stimulation of  **PKC**  activity in hippocampal slices either with phorbol esters or with the mGluRs specific agonist trans 1 amino cyclopentanedicarboxylic acid t ACPD decreases alphaCaMKII binding to  **NMDA**  receptor complex.  Ref: 11104776 J Biol Chem, 2001
